# Supplementary material for: Genome-Wide Association Analysis of Fruit Shape-Related Traits in Areca catechu
Source: Int J Mol Sci. 2023 Feb 28;24(5):4686. doi: 10.3390/ijms24054686 (PMC10003628; doi:10.3390/ijms24054686)
Supplement: Supplementary file 1 [file ijms-24-04686-s001.zip › Additional file Figure.pdf]

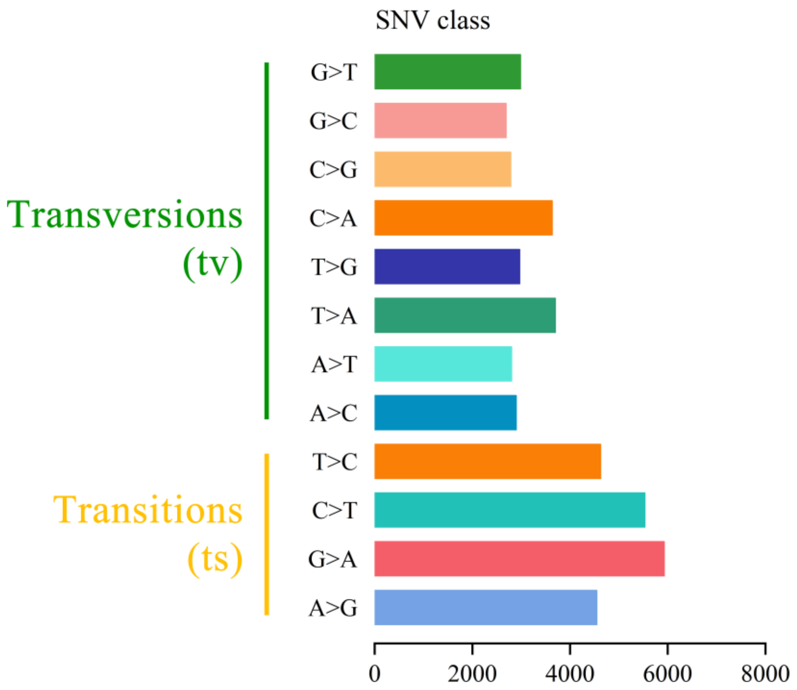

Figure S1. The number of Transitions (ts) type and Transversions (tv) type SNPs.

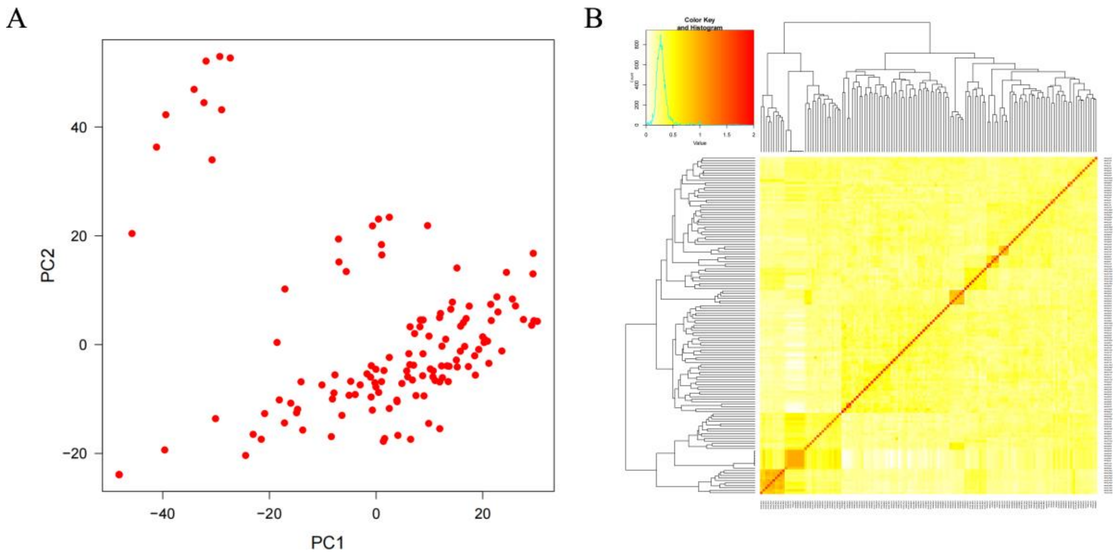

Figure S2. Genetic relationship analysis. (A) principal component analysis. (B) Kinship heat map.

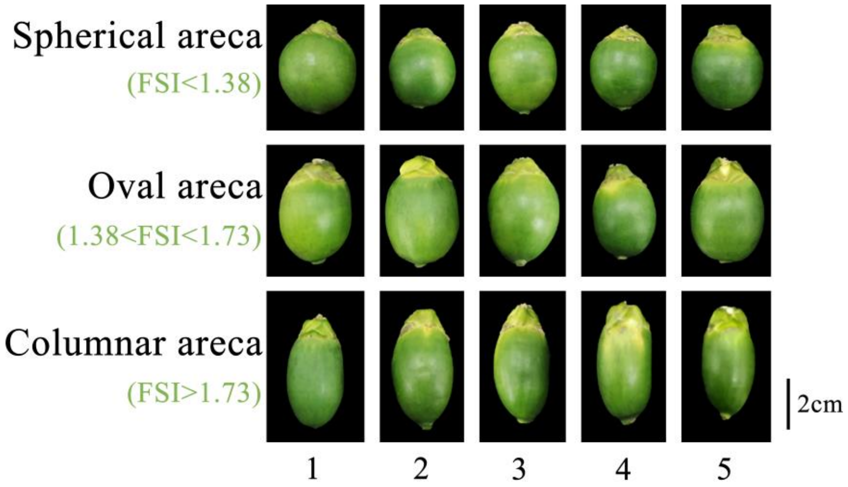

Figure S3. Fruit morphology of different areca germplasms. FSI: fruit shape index.
